# A Smartphone-Enabled Imaging Device for Chromotropic Acid-Based Measurement of Nitrate in Soil Samples

**DOI:** 10.3390/s23177345

**Published:** 2023-08-23

**Authors:** Veerabhadrappa Lavanya, Anshuman Nayak, Partha Deb Roy, Shubhadip Dasgupta, Subhadip Dey, Bin Li, David C. Weindorf, Somsubhra Chakraborty

**Affiliations:** 1 Agricultural and Food Engineering Department, Indian Institute of Technology, Kharagpur 721302, India; lavanya810512013@gmail.com (V.L.); anshumannayak290698@gmail.com (A.N.); partha.slg09@gmail.com (P.D.R.); sd_g@hotmail.com (S.D.); sdey23@agfe.iitkgp.ac.in (S.D.); 2 ICAR-Indian Institute of Water Management, Bhubaneswar 751023, India; 3 Department of Agricultural Chemistry and Soil Science, Bidhan Chandra Krishi Viswavidyalaya, Mohanpur 741252, India; 4 Department of Experimental Statistics, Louisiana State University, Baton Rouge, LA 70803, USA; bli@lsu.edu; 5 Department of Earth and Atmospheric Sciences, Central Michigan University, Mount Pleasant, MI 48859, USA; weind1dc@cmich.edu

**Keywords:** soil, nitrate, smartphone, imaging device, kriging

## Abstract

In this study, a novel chromotropic acid-based color development method was proposed for quick estimation of soil nitrate (NO_3_^−^). The method utilized a 3D printed device integrated with the rear-end camera of a smartphone and a stand-alone application called SMART NP. By analyzing the mean Value (V) component of the sample’s image, the SMART NP provides instant predictions of soil NO_3_^−^ levels. The limit of detection was calculated as 0.1 mg L^−1^ with a sensitivity of 0.26 mg L^−1^. The device showed a % bias of 0.9% and a precision of 1.95%, indicating its reliability. Additionally, the device-predicted soil NO_3_^−^ data, combined with kriging interpolation, showcased spatial variability in soil NO_3_^−^ levels at the regional level. The study employed a Gaussian model of variogram for kriging, and the high Nugget/Sill ratio indicated low spatial autocorrelation, emphasizing the impact of management factors on the spatial distribution of soil NO_3_^−^ content in the study area. Overall, the imaging device, along with geostatistical interpolation, provided a comprehensive solution for the rapid assessment of spatial variability in soil NO_3_^−^content.

## 1. Introduction

Effective soil and water nitrate management is of paramount importance due to its significant impact on both environmental and human health. Notably, NO_3_^−^, a common form of nitrogen, is an essential nutrient for plant growth [1]. However, excessive NO_3_^−^ levels in soil and water can lead to numerous detrimental consequences. Agricultural activities, such as excessive fertilizer application and poor irrigation practices, contribute to elevated NO_3_^−^ levels in soil and water systems [2]. When NO_3_^−^ leaches into water bodies, it can lead to eutrophication, causing algal blooms that deplete oxygen levels and disrupt aquatic ecosystems. Moreover, when contaminated water is consumed, particularly by infants and pregnant women, it can result in methemoglobinemia or “blue baby syndrome”, a condition that impairs the oxygen-carrying capacity of blood. Therefore, it is crucial to implement effective soil NO_3_^−^ management strategies to protect ecosystems, preserve water quality, and safeguard human health.

Several methods are available for measuring soil NO_3_^−^ levels, each with its own advantages and limitations. The most commonly used methods include colorimetry, ion-selective electrode (ISE) analysis, and chromatographic techniques [3,4,5,6]. Chemical extraction methods, such as KCl extraction, provide a reliable and cost-effective means of determining soil NO_3_^−^ content [7]. However, they require time-consuming sample preparation and may underestimate NO_3_^−^ levels in certain soil types. ISE analysis offers rapid and precise measurements, but it requires specialized equipment and expertise. Chromatographic techniques, such as ion chromatography and high-performance liquid chromatography, can offer accurate quantification of NO_3_^−^, even in complex soil matrices [8]. These techniques offer high sensitivity and selectivity but are often expensive and require sophisticated instruments and trained personnel. Overall, the choice of method depends on the specific requirements of the study, considering factors such as cost, time, accuracy, and available resources. Advances in technology, including smartphone-integrated devices, offer promising alternatives, combining convenience, affordability, and accuracy for on-site NO_3_^−^ measurements.

The Integration of smartphone technology with image processing techniques has opened up new avenues for predicting various soil and water parameters [9,10]. Soil image processing using smartphones involves capturing images of soil samples and analyzing them through dedicated applications or software. These images can be processed to extract valuable information related to soil texture [9], organic matter content [10], moisture levels [11], soil pH [12], water contaminants [13], and even nutrient concentrations [14,15,16]. By leveraging machine learning algorithms, these smartphone-based systems can generate predictive models that correlate image features with soil and water parameters of interest [9,10]. This approach offers several advantages, including accessibility, cost-effectiveness, and real-time analysis capabilities [17]. Moreover, handheld smartphone spectrophotometry systems have shown potential for real-time detection of Cu and Fe in water [18]. These enable researchers, farmers, and environmentalists to rapidly assess soil quality and make informed decisions regarding nutrient management, irrigation practices, and land use planning. However, challenges remain in standardizing image acquisition protocols, ensuring accuracy across different smartphone models, and establishing robust calibration models for accurate parameter predictions [19]. Continued advancements in image processing algorithms, coupled with smartphone capabilities, hold great potential for revolutionizing soil analysis, promoting sustainable land management practices, and enhancing agricultural productivity.

Building upon our previous research [16], we have further advanced the capabilities of the smartphone-integrated imaging device, along with the novel “SMART NP” mobile application, for the rapid estimation of soil NO_3_^−^ levels. In our previous study, we successfully utilized different coloring agents to visually express the concentration of soil and water NO_3_^−^ and PO_4_^3−^ and subsequently captured images of the samples for color parameter extraction and predictive modeling. However, in previous attempts, the conventional phenoldisulphonic acid method failed to generate noticeable color distinctions in captured images that corresponded to varying concentrations of NO_3_^−^ in soil and water [16]. Hence, in this new research, another convenient chromotropic acid (CTA)-based color development method [20] was tested with the smartphone-integrated device, specifically tailored for predicting soil NO_3_^−^ levels. The ultimate goal of this research was to provide a user-friendly and cost-effective tool for farmers, researchers, and environmentalists to assess NO_3_^−^ levels on-site, enabling informed decision-making for nutrient management and sustainable agricultural practices. 

The objectives of this research were to: (1) Optimize and validate the smartphone-integrated imaging system for the rapid and accurate estimation of soil NO_3_^−^ levels using an advanced CTA-based color development method and (2) evaluate system-predicted soil NO_3_^−^ in conjunction with geostatistical interpolation for rapid evaluation of soil NO_3_^−^ spatial distribution at the regional level in Eastern India with comparison to laboratory-derived data. We hypothesized that the smartphone-integrated imaging device, coupled with the advanced CTA-based color development methods, would provide rapid and accurate estimation of soil NO_3_^−^ levels. Additionally, we anticipate that the device-predicted soil NO_3_^−^ values, when combined with geostatistical interpolation techniques, will effectively capture the spatial distribution of soil NO_3_^−^ levels at the regional scale in Eastern India.

## 2. Materials and Methods

### 2.1. Setup of the Imaging Device

**Figure 1:**
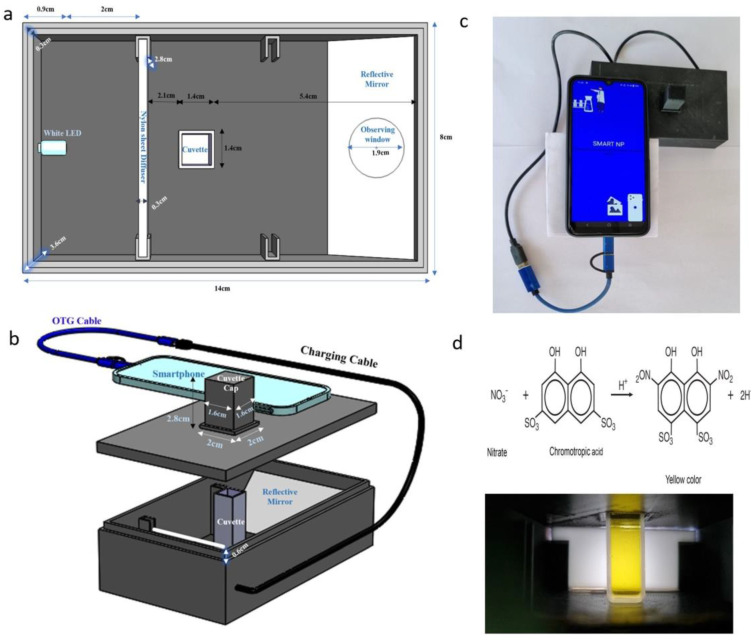
(**a**) Internal structural diagram of the proposed device, (**b**) the whole assembly with a smartphone, (**c**) the smartphone application, and (**d**) chromotropic acid method reaction and resultant yellow colored solution in the cuvette.

The smartphone-integrated imaging system was constructed with a 3D-printed black box measuring 14 cm × 8 cm × 4.2 cm [16]. The box was designed using SOLIDWORKS version 2022 (Waltham, MA, USA) and printed using black 1.75 mm polylactic acid filament. The printing process was carried out using an ENDER 3 3D printer (Shenzhen Creality, Shenzhen, China) (Figure 1) [16]. This device seamlessly connects to a smartphone, functioning as a plug-and-play device, and its internal white LED light is powered by the smartphone’s battery using an ONCRO^®^ Blue 2 in 1 On-The-Go type C USB adapter cable. To avoid intense light on the soil extract solution, a diffuser made of a nylon sheet (6.8 cm × 2.8 cm × 0.3 cm) was positioned 2 cm away from the white LED light source. The soil extract was carefully poured into a cuvette with dimensions of 4.4 cm × 1.2 cm × 1.2 cm. The cuvette had a 1 cm optical path length and was positioned at the center of the diffuser. A reflective plane surface, in the form of a 2 mm thick mirror, was set at a 45° angle, resulting in an optical path length of 3.7 cm between the cuvette and the observing hole, which had a diameter of 1.9 cm. To shield from external light interference, a cuvette cap (2.8 cm × 1.6 cm × 1.6 cm) was used to cover the cuvette containing the solution.

To capture sample images, an HONOR-20i smartphone was employed, featuring a metal-oxide semiconductor (CMOS) sensor with specifications of 24 MP, 1920 × 1080 pixels, and an f/1.8 aperture. The smartphone was equipped with a non-removable 3400 mAh lithium polymer battery that supported 5 V/2 A 10 W fast charging. The provision of the diffuser ensured that the value (V) component of the smartphone-captured images remained unsaturated, allowing for a relatively linear variation of V values with fluctuations in NO_3_^−^ concentration in the extract [14]. The combined weight of the entire device, including the smartphone, was approximately 250 g, making it suitable for field use. For more details on the device, see [16].

### 2.2. Laboratory Extraction and Device Calibration

**Figure 2:**
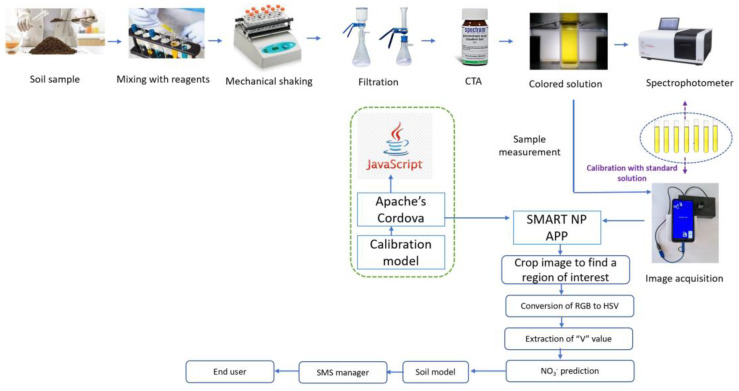
The process flow diagram of the entire proposed approach.

To ensure minimal contamination, all glassware used for laboratory analyses was thoroughly washed with ethanol (80%) and deionized water. The prediction of NO_3_^−^ in soil samples was carried out via the CTA method, utilizing colorimetric measurements [20]. Initially, 0.036 g potassium nitrate was dissolved in 1 L of deionized water to prepare 50 mg L^−1^ NO_3_^−^ stock solution. To facilitate color development, a concentrated H_2_SO_4_ solution was used to prepare a 0.1% stock solution of CTA disodium salt. This involved dissolving 1.84 g of practical grade CTA in 1 L of reagent grade H_2_SO_4_, with a specific gravity of 1.84 g cm^−3^. Following this, a 0.01% working solution of CTA was created by diluting 100 mL of the 0.1% stock solution and 10 mL of concentrated HC1 to 1 L with concentrated H_2_SO_4_. To prepare soil extracts, a 5 g soil sample was weighed in a Mettler Toledo analytical balance (Mettler Toledo, Columbus, OH, USA) and combined with 25 mL of reagent A (CuSO_4_ mixed with AgSO_4_) in an Eltek orbital mechanical shaker (Elektrocraft India Pvt. Ltd., Vasai-Virar, MH, India) for 15 min. Subsequently, the soil solutions were further mechanically shaken for an additional 5 min after adding 0.04 g of calcium hydroxide and 0.1 g of magnesium carbonate. Afterward, the soil solutions were left undisturbed for a duration of 10 min and subsequently filtered using Whatman filter paper No. 42. Finally, the yellow CTA-NO_3_^−^ complex was made by mixing 3 mL of soil NO_3_^−^ extract and 7 mL of the 0.01% CTA working solution. The prepared NO_3_^−^ solutions were then subjected to batch-wise spectrophotometric analysis using a UV-VIS spectrophotometer (Model UV-3200, LabIndia, Mumbai, MH, India) at 430 nm [21] (Figure 2).

Calibration of NO_3_^−^ in soil samples involved creating 12 standard solutions (0, 0.5, 1.5, 2.5, 3.5, 4.5, 5.5, 6.5, 7.5, 8.5, 9.5, and 10 mg L^−1^) by diluting the NO_3_^−^ stock solution with deionized water. Next, CTA was added to the standard solutions, and photographs were taken using the smartphone-integrated device in conditions of controlled and uniform illumination, set at 12 Lux, with five replications. An HTC digital Lux meter was used to measure the illumination. The mean Value (V) component of the Hue-Saturation-Value (HSV) color model was calculated from the soil extract images and subsequently used to calibrate soil NO_3_^−^ [16]. The calibration equation for NO_3_^−^ in soil covered a range of 0.0–10.0 mg L^−^^1^. In this study, the limit of detection (LOD) was calculated using Equation (1) [16]:

(1) $$\mathrm{LOD}=\frac{3.3SD}{\sigma }$$

where SD represents the standard deviation of the device’s results, while σ represents the device’s sensitivity. Factor 3.3 is applied under the assumption that the noise adheres to a normal distribution. Sensitivity was calculated as the ratio of the change in the output of the device (soil NO_3_^−^ concentration) to the corresponding change in the input value (average V value) per [22]. Moreover, % relative standard deviation (RSD), which is an indicator of variability or dispersion of the measurements obtained, and % bias were computed using Equations (2) and (3) [14], respectively:

(2) $$\%\;\mathrm{RSD}=\frac{SD}{Mean}\times 100$$

(3) $$\%\;\mathrm{bias}=\frac{\left(Known\;{NO}_{3}^{-}\;concentration-Mean\;{NO}_{3}^{-}\;concentration\right)}{Known\;{NO}_{3}^{-}\;concentration}\times 100$$

For both % RSD and % bias computation, six NO_3_^−^ solutions (0.25, 1.25, 2.75, 3.75, 5.75, and 8.75 mg L^−1^) were arranged and subsequently imaged in five replicates. The six concentrations were carefully selected to represent the entire calibration range of 0.0–10.0 mg L^−1^. In addition to testing with the HONOR-20i, two more smartphone cameras with different resolutions (Redmi 12 Pro and Vivo Y1S with 50 and 13 MP, respectively) were tested for predicting soil NO_3_^−^ using the same methodology and subsequently compared their results.

### 2.3. Image Analysis and SMART NP Development

The mobile app called “SMART NP” was created and designed to measure the levels of NO_3_^−^ in soil samples [16]. It utilizes the V component of the color space model HSV to quantify the NO_3_^−^ concentrations accurately. The HSV color model is a representation of colors based on three components: hue (H), saturation (S), and value (V) [23]. The conversion from RGB (Red-Green-Blue) color space to HSV color space involves a series of mathematical operations as follows [24]:

Step 1: Normalize the RGB values: divide the RGB values by 255 to bring them into the range of 0 to 1.

R′ = R/255 G′ = G/255 B′ = B/255(4)

Step 2: Calculate the maximum (max) and minimum (min) values among R′, G′, and B′:

max = max(R′, G′, B′) min = min(R′, G′, B′) (5)

Step 3: Calculate the hue (H) component:

If max = min, then H is undefined (the color is achromatic, i.e., a shade of gray)(6)

Otherwise, if max = R′, then H = 60 × (G′ − B′)/(max − min) (with the possibility of adding 360 to ensure the value is positive) (7)

Otherwise, if max = G′, then H = 60 × (2 + (B′ − R′)/(max − min))(8)

Otherwise, if max = B′, then H = 60 × (4 + (R′ − G′)/(max − min)) (9)

Step 4: Calculate the saturation (S) component:

If max = 0, then S = 0 (the color is achromatic) (10)

Otherwise, S = (max − min)/max (11)

Step 5: Calculate the value (V) component:

V = max(12)

Notably, the above conversion assumes RGB values in the standard RGB (sRGB) color space, commonly used in digital displays and images. Different RGB color spaces may require different conversion formulas. The HSV model’s V component is particularly suitable for the SMART NP application [16]. SMART NP is a novel mobile app created with Apache Cordova (v10.0.0) [25], designed to analyze soil NO_3_^−^ without requiring an internet connection. In the soil sample mode, users enter details, select, or capture an image, correct image orientation using EXIF data, and resize it to 1080p. Next, the app crops the image to isolate and identify the specific region of interest (ROI) [16]. RGB to HSV conversion is applied to the ROI, and the average V value is extracted. The soil NO_3_^−^ is predicted using the average V value. The system-predicted results can be sent to the end users or farmers via SMS, WhatsApp, and email. Notably, the code base of SMART NP supports Android, iOS, Windows, Blackberry, webAssembly, and scalable JavaScript (Figure 2).

### 2.4. Sample Collection and Device Performance Validation

To validate the device, a total of 485 surface soil samples (0–20 cm) were collected from the Coastal Saline Zone, Red and Lateritic Zone, Hilly Zone, Old Alluvial Zone, and New Alluvial Zone in Eastern India [26]. These samples were collected from six districts, namely Nadia (n = 112), East Medinipur (n = 74), Jhargram (n = 97), Birbhum (n = 95), South 24 Parganas (n = 50), and Darjeeling (n = 58), and represented three soil orders (Alfisols, Entisols, and Inceptisols) [27] and four soil parent materials (granite-gneiss, old alluvium, recent alluvium, and peninsular colluvium) [26]. After the rice harvest, in November-December, the rice field soil samples were collected in a 5 km grid. The sampling process was undertaken in both rainfed and irrigated agricultural soils across small, medium, and large land holdings. Georeferencing was performed using a Garmin E-Trex global positioning system receiver (Garmin, Olathe, KS, USA). Next, the samples underwent a series of steps, including air-drying, grinding, sieving through a 2-mm sieve, NO_3_^−^ extraction, and CTA-based color development in preparation for subsequent imaging analysis. These soil samples were analyzed using both the standard spectrophotometric method and the device-based method depicted in Section 2.2.

The statistical analyses were conducted using R 4.2.2 [28]. The association between spectrophotometer-reported values and the average device-predicted values was tested. Additionally, to evaluate the correlations between the individual replicate results obtained from the device, both Pearson’s correlation test and Kendall’s correlation test were utilized. Pearson’s correlation test employed the “cor” function, while Kendall’s correlation test utilized the “cor.test.kendall()” function from the Kendall package in R [29]. Kendall’s test, being a rank-based measure of association, is particularly suitable when the data does not conform to a bivariate normal distribution. Moreover, the analysis included the application of the roughness of concomitant ranks (RCR) test [30], which is a rank-based test of association. This test was employed to examine both linear and nonlinear dependencies between two numeric vectors. Importantly, the RCR test demonstrates greater sensitivity in detecting nonlinear associations between the vectors compared to Kendall’s test and other conventional tests.

### 2.5. Soil NO_3_^−^ Spatial Variability Mapping Using the Device

Geospatial and geostatistical analyses were carried out using ArcGIS 10.3.1 (ESRI, The Redlands, CA, USA). For predicting soil NO_3_^−^ concentrations at unsampled locations and creating maps, Kriging interpolation was employed [31]. These maps displayed the predicted soil NO_3_^−^ values generated by the device and were subsequently compared to maps created using lab-reported soil NO_3_^−^ values. Variograms were used to assess the statistical dependence and spatial autocorrelation for each indicator. The best-fitted model (such as circular, spherical, exponential, Gaussian, or linear) for soil NO_3_^−^ was determined using the ESRI ArcGIS Geostatistical Analyst (The Redlands, CA, USA), predicting for non-sampled areas [32].

## 3. Results and Discussion

### 3.1. NO_3_^−^ Calibration Models

**Figure 3:**
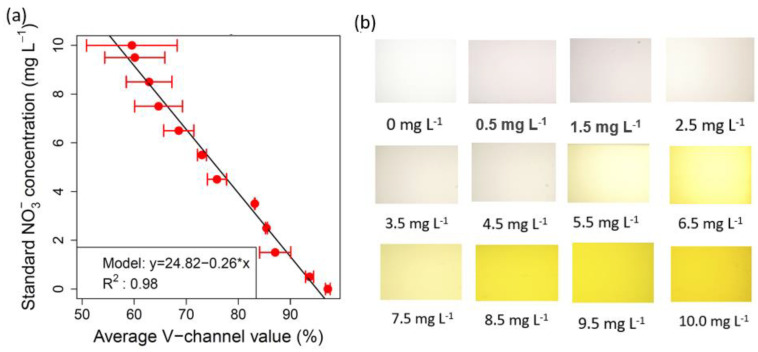
Plots showing (**a**) the calibration for soil NO_3_^−^ (measured using spectrophotometer) using the device-extracted average V values, and (**b**) colored images of the chromotropic acid-treated standard NO_3_^−^ solutions taken by the smartphone camera.

As the reagents reacted with the NO_3_^−^, the solutions gradually changed from pink to bright yellow, with the color intensity increasing with higher NO_3_^−^ concentrations. Figure 3 presents the calibration models for soil NO_3_^−^, showcasing images of the standard NO_3_^−^ samples. Soil NO_3_^−^ calibration plot showed a consistent linear decreasing trend in the V-value as the NO_3_^−^ concentrations increased. The calibration relationship for soil NO_3_^−^ demonstrated high precision, with an R^2^ value of 0.98. The soil NO_3_^−^ calibration equation is given as Equation (13):

Soil NO_3_^−^ = 24.82 − 0.26 V-value (13)

Subsequently, the equation was employed to predict NO_3_^−^ in real soil samples. Notably, variations in V channel values for replicate readings, particularly in cases of higher nitrate concentrations (>6 mg L^−1^) can be attributed to the sensitivity to color intensity, device and lighting consistency, sample homogeneity, and some limitations in the optical components. While the smartphone-integrated imaging system is designed to capture color changes accurately, subtle variations in lighting conditions, as well as the inherent variability in the reaction kinetics of color development, might have led to slightly different color intensities across replicate readings [33,34]. While the 3D-printed device and smartphone imaging system provide a controlled environment for capturing images, there could be minor inconsistencies in factors such as the positioning of the cuvette, alignment of the mirror, and stability of the smartphone’s white LED light source. These factors might have introduced slight differences in the illumination of the samples, affecting the recorded color values. Additionally, the optical components, including the mirror and diffuser, might have introduced subtle distortions or attenuations in the captured images [35]. Another reason could be the non-homogeneous distribution of NO_3_^−^ ion, particularly at higher concentrations. Nevertheless, the two other tested mobile devices with variable camera specifications also produced comparable results (Table 1). Table 2 shows a comparison of the performance for measuring analytes in soil and water using smartphone-based methods, as reported by researchers.

### 3.2. Device Characteristics

Within the tested range of 0 to 10 mg L^−1^, the pink-yellow color intensity in the CTA method remained stable within a reaction time range of 10–60 min for NO_3_^−^. These results were consistent with the findings of [16], who observed color stability within the yellow range for NO_3_^−^ during the 10–60 min timeframe using the phenoldisulphonic acid method. To ensure precise measurements, the device incorporated LED light, a cover lid, and a cuvette cap, providing consistent illumination and contributing to favorable precision with an RSD of 1.95%. This precision exceeds that reported by [14] (2.05%). The inter-day precision achieved by the proposed device was 1.50%, outperforming the precision of 1.75% reported by [16]. The exhibited sensitivity of the imaging device was 0.26 mg L^−1^. The LoD for NO_3_^−^ was calculated as 0.1 mg L^−1^. Additionally, the % bias for the NO_3_^−^ calibrations was determined to be 0.9%, representing a significant improvement compared to the phenoldisulphonic acid-based protocol [16].

Notably, CMOS sensors in smartphones are highly advantageous for smartphone-based chemical analysis due to their fast readout speed, low noise levels, high sensitivity, wide dynamic range, and seamless integration [36]. The fast readout speed enables quick measurements, reducing analysis time, while the low noise produces accurate color reproduction and better image quality. The high sensitivity allows for the detection of subtle color or absorbance changes, while the wide dynamic range captures a broad range of intensities. Moreover, the compact size and integration with smartphones make CMOS sensors convenient, portable, and accessible for a wide range of analytical applications, making chemical analysis more efficient and widely available [37]. Notably, the proposed protocol allows for a sample throughput of approximately 32 samples h^−1^, as the designed setup can analyze eight air-dried and sieved soil samples within a 15-min timeframe.

### 3.3. Real Soil Test Performance

**Figure 4:**
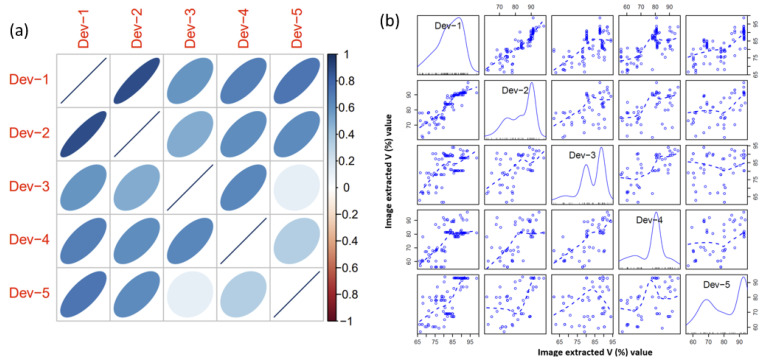
(**a**) Plot showing the correlation between the device’s five replicates V-value readings for soil NO_3_^−^, and (**b**) matrix of scatterplots indicating the relationship between the device’s readings (image extracted V value) for NO_3_^−^ in soil. Note that Dev-1, 2, 3, 4, and 5 indicated five replicate V-value readings from the device. In the matrix, the blue dashed curves represent a nonlinear smoothing method via local polynomial regression or the locally estimated scatterplot smoother (LOESS). Additionally, the diagonal plots show the computed density plots for each device replicate.

**Figure 5:**
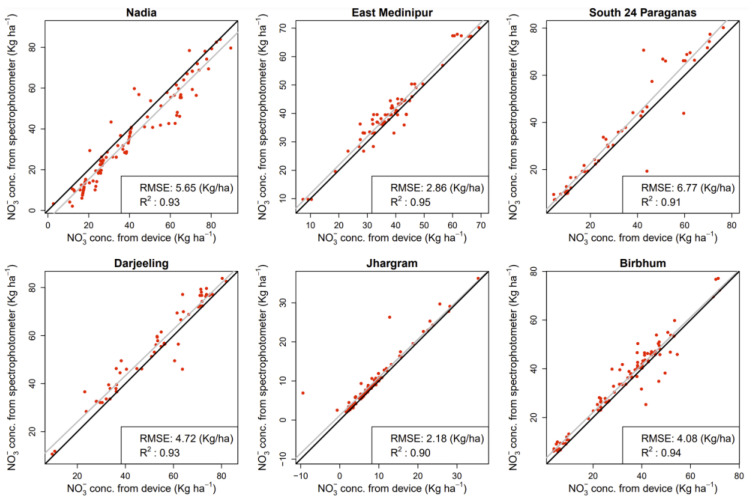
Correlation between mean device-predicted values vs. mean conventional spectrophotometer-based results for NO_3_^−^ in soil samples. The grey line and the solid black line represent the fitted linear regression line and the 1:1 line, respectively.

To evaluate the device’s applicability, soil (n = 485) samples were collected and tested. For soil samples, the proposed device measured NO_3_^−^ in five replications. The results exhibited a strong Pearson’s correlation between the replicates of the device’s readings for soil extracts (Figure 4a). The *p*-values associated with these correlations were extremely small, approaching zero in most cases, suggesting a high level of confidence in the presence of linear relationships between the replicate readings. Moreover, based on the *p*-values from Kendall’s correlation test, there were strong and significant correlations among most pairs of replicate readings. All replicates exhibit significant correlations with each other, suggesting a monotonic relationship between their readings. Based on the *p*-values from the RCR test, there was strong evidence of significant ordinal relationships among all pairs of replicate readings. From the scatterplot matrix plot with added nonlinear smoother (Figure 4b), obvious positive associations were found between the replicates for soil NO_3_^−^ measurement. No extreme outliers were observed for the replicates. These findings highlighted the consistent and comparable nature of device measurements in the presence of natural soil samples. However, to further enhance robustness, future datasets should include additional soil samples for validation purposes. The average device-predicted values and the spectrophotometer-based standard laboratory estimation for NO_3_^−^ displayed a significantly high correlation. A high coefficient of determination was obtained for soil (R^2^ of 0.90 to 0.95) samples (Figure 5). Furthermore, an independent samples *t*-test was executed using 30 randomly selected real soil samples to explore the potential disparity between the outcomes acquired through the novel device and the established spectrophotometer. The null hypothesis postulated that no substantial disparity would exist between the data originating from the two methodologies. The outcomes revealed that the mean measurements of NO_3_^−^ in soil samples obtained from the device and the spectrophotometer exhibited no statistically significant dissimilarity (*p*-value > 0.05). These findings underscored the feasibility of the introduced device in practical applications. Note that the kg ha^−1^ was used to evaluate nutrient availability, guide fertilization decisions, and manage nutrients effectively. This standardized unit enables reliable comparisons of soil nutrient levels across diverse locations, soil types, and management approaches. Furthermore, kg ha^−1^ offers practicality and scalability for agricultural operations, facilitating seamless implementation and compatibility with fertilizer recommendations aligned with crop nutrient requirements and yield objectives.

### 3.4. Spatial Variability Mapping of Soil NO_3_^−^

**Figure 6:**
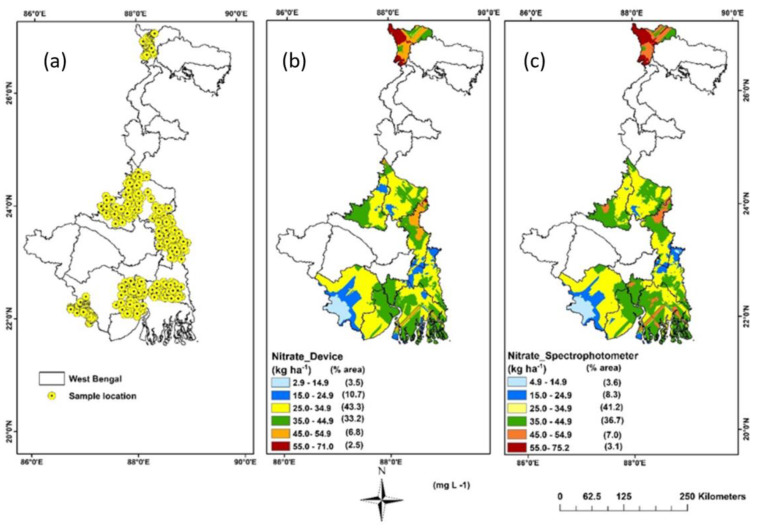
Figure showing (**a**) soil sampling locations in India, (**b**) kriging map using the device-predicted values, and (**c**) kriging map using the conventional laboratory analysis.

A comprehensive evaluation and comparison of various kriging models was conducted to estimate soil NO_3_^−^ values at unsampled locations (Figure 6). The soil NO_3_^−^ data obtained from the device was subjected to kriging, with the intention of enabling future soil research to directly employ smartphone-based proximal sensor predicted results as input for the predictive model. The selection of the most suitable variogram model was based on the Nugget/Sill ratio results. Table 3 summarizes the regression equations used for the kriged dataset. Besides, similar kriging models and functions were used with laboratory soil NO_3_^−^ data for visual comparison (Figure 6). Notably, among all variogram models, the Gaussian model yielded the lowest mean standard error across the predicted surface and optimally predicted soil NO_3_^−^ concentrations, with the nugget, partial sill, and range parameters achieving desirable values.

The device predicted soil NO_3_^−^ content exhibited a general increase from west to east, ranging from 2.9 to 71 kg ha^−1^, with the exception of some small patches in the eastern region. The kriged maps using laboratory data also reflected a similar increasing trend from west to east. The higher concentration of soil NO_3_^−^ in the eastern part can be attributed to intensive agricultural practices, including the use of high-yielding rice varieties and a higher rate of nitrogenous fertilizer applications [38]. Conversely, the lower availability of soil NO_3_^−^ in the western part can be attributed to factors such as lower soil pH, higher mean temperature during the warmest and wettest quarters, and a larger area of bare soil due to lower precipitation [39]. This variability can also be attributed to the undulating terrain and non-uniform management of fallow land with degraded vegetation cover, leading to visible differences in surface soil properties over short distances. For both maps, the northern hilly region indicated a higher level of soil NO_3_^−^ content since grassland/forest land use structure in the hills has a better capability for soil conservation and retaining nutrients than other land use types [40]. The majority of the mapped area (43.3%) using device-predicted values displayed soil NO_3_^−^ concentrations ranging from 25.0 to 34.9 kg ha^−1^, followed by soil NO_3_^−^ ranges of 35.0 to 44.9 kg ha^−1^ (33.2%), 15.0 to 24.9 kg ha^−1^ (10.7%), 45 to 54.9 kg ha^−1^ (6.8%), 2.9 to 14. 9 kg ha^−1^ (3.5%), and 55.0 to 71.0 kg ha^−1^ (2.5%). The spatial variability maps generated from laboratory measurements of soil NO_3_^−^ exhibited a similar trend, with soil NO_3_^−^ ranges of 25.0–34.9, 35.0 to 44.9 kg ha^−1^, 15.0 to 24.9 kg ha^−1^, 45 to 54.9 kg ha^−1^, 4.9 to 14.9 kg ha^−1^, and 55.0 to 75.2 kg ha^−1^, representing 41.2%, 36.7%, 8.3%, 7%, 3.6%, and 3.1% of the mapped area, respectively. Generally, a higher Nugget/Sill ratio indicates that stochastic factors such as fertilizer application, farming practices, cropping systems, and other anthropogenic activities primarily contribute to spatial variability [41]. Conversely, a lower ratio indicates that structural factors, such as climate, parent material, soil attributes, relief, and other natural features, play a crucial role. The Nugget/Sill ratios of 0.81 and 0.85 for the device and laboratory data, respectively, indicate a low level of spatial autocorrelation, highlighting the importance of management factors in determining the spatial variability of soil NO_3_^−^ content in the study area.

### 3.5. Practical Utility of the Proposed Approach

The utilization of smartphone-integrated imaging devices, coupled with advanced CTA methods, presents a promising solution for resource-poor farmers in various agro-climatic zones. This technology offers the potential to enhance soil NO_3_^−^ prediction and subsequent mapping, thereby enabling farmers to make informed decisions regarding nutrient management strategies. Moreover, the farmer could compare this result with historical data, crop nutrient requirements, and target levels for optimal growth. In resource-poor conditions, where access to expensive laboratory testing and sophisticated equipment is limited, the smartphone-integrated imaging device provides a cost-effective and user-friendly alternative [9,10]. Farmers can easily capture soil data using their smartphones, eliminating the need for specialized training or technical expertise. By implementing this technology in their farming practices, resource-poor farmers can gain valuable insights into the spatial distribution of soil NO_3_^−^ content. The kriging models, evaluated and compared through the comprehensive study, offer accurate estimations of soil NO_3_^−^ values at unsampled locations. This information allows farmers to identify areas with high or low soil NO_3_^−^ concentrations, enabling them to tailor their nutrient management strategies accordingly.

For instance, the study demonstrated a general increase in soil NO_3_^−^ content from west to east, with the eastern region exhibiting higher concentrations due to intensive agricultural practices. By utilizing the smartphone-integrated imaging device, farmers can identify these high NO_3_^−^ zones and adopt appropriate management practices, such as adjusting fertilizer application rates or employing nitrogen-fixing cover crops [42], to optimize nutrient utilization and minimize environmental impacts. Conversely, resource-poor farmers in the western region, characterized by lower soil NO_3_^−^ availability, can leverage this technology to pinpoint areas with nutrient deficiencies. By understanding the factors contributing to lower NO_3_^−^ levels, such as soil pH, temperature, and precipitation, farmers can implement targeted interventions to enhance soil fertility. This may include soil amendments, irrigation management, or the selection of crop varieties adapted to specific soil conditions [43].

Furthermore, the device’s ability to inform spatial variability maps provides resource-poor farmers with valuable information about the heterogeneity of soil NO_3_^−^ content within their fields. In the future, farmers will be informed about the variability through color-coded maps generated by the application, showcasing areas with higher or lower soil NO_3_^−^ levels. This knowledge will allow them to implement site-specific management strategies, optimizing input utilization while minimizing costs. By precisely identifying areas with different soil NO_3_^−^ ranges, farmers can tailor their fertilizer applications, saving resources and reducing the risk of nutrient runoff or leaching. The accessibility and affordability of smartphone technology make this approach particularly suitable for resource-poor farmers. With the increasing penetration of smartphones, even in rural areas, farmers can easily access and utilize this technology. The user-friendly interface and simplified data acquisition process eliminate barriers that may have hindered the adoption of traditional soil testing methods. Farmers can also benefit from expert advice and extension services to interpret results and develop effective nutrient management plans tailored to their specific conditions.

In field soil testing, the process involves soil sampling and obtaining small particles (<2 mm) through air-drying and sieving. Farmers should collect soil samples using a consistent protocol [44]. To ensure representativeness, samples should be collected from multiple locations across the field, typically following a zigzag pattern or grid layout. For surface soil samples, the top 0–15 cm layer is usually sampled, as it is most relevant to plant nutrient availability. Each individual sample should be a composite of subsamples collected within a specific sampling area. The number of subsamples can vary, but a common practice is to collect around 10–15 subsamples from each sampling area. Subsamples should be mixed thoroughly to form a composite sample representative of that specific area. Besides, farmers should avoid collecting samples from unusual spots, such as areas near trees, fence lines, or other sources of potential contamination.

If the soil is already dry, sieving alone is sufficient. Conversely, for wet soil, proper drying is necessary before proceeding with testing. Efficient sample preparation is a critical aspect of any field-testing method. However, there are already portable soil testing kits available in the market that address the challenges of sample preparation and reagent handling [45]. These kits are designed to streamline the testing process by providing pre-measured reagents, simplified sample processing steps, and user-friendly interfaces. These kits are specifically designed to cater to field conditions and the needs of resource-poor farmers. For instance, several companies offer compact and portable soil testing solutions that come equipped with pre-packaged reagents, easy-to-use sample processing steps, and user-friendly instructions. These kits have been successfully utilized in various field settings, allowing users to perform soil testing without the need for extensive laboratory equipment. These examples demonstrate the feasibility of conducting on-site soil testing with relatively minimal effort.

Alternatively, portable soil dryers are recently available to expedite the drying process, utilizing propane or natural gas to dry soil samples within 30 min. Desiccant bags comprising moisture-absorbing material can also help in on-site drying. Additionally, solar dryers and fan-assisted air-drying methods are viable options for processing field samples. In laboratory testing, the proposed method utilizes a smartphone as the detector, requiring basic sample preparation and extraction. Two extraction methods commonly used in the field are shaking and squeezing. Conversely, in the shaking extraction method, the soil sample mixed with a suitable extractant solution is placed in a sealed bag. Next, the bag is then vigorously shaken for thorough mixing and subsequent extraction of the liquid portion. Alternatively, squeezing extraction involves adding the extractant solution directly to the sealed bag containing soil and gently squeezing or kneading to extract the liquid. While these field methods may not match the precision of laboratory-grade techniques, they eliminate the need for advanced instrumentation, making them suitable for rudimentary laboratories or those in developing countries. Furthermore, the utilization of a smartphone’s battery enhances its portability and convenience in the field, eliminating the requirement for external power sources. The larger battery capacity of the smartphone allows the whole setup to operate for extended periods without the need for recharging.

To provide rapid screening of soil NO_3_^−^ concentrations after sample preparation, the proposed device with CTA protocol is optimized for field use while acknowledging that it does not aim to substitute conventional soil testing protocols. Unlike contemporary colorimetric methods featuring spectrum-based investigation and point-based measurements, this research introduces a camera-based method for capturing the complete absorption features. Notably, the device selects a relevant area of interest within the sample-containing cuvette using the SMART NP application and subsequently calculates the mean V value using multiple pixels after equalizing the color histogram for standardized and reliable image feature extraction. Consequently, SMART NP offers a more reliable estimate of the analyte than some contemporary open-source applications that rely on single-point measurements [16]. Thus, the device accurately predicts NO_3_^−^ concentration using the mean V value via a pre-trained model. Admittedly, while technology reduces the need for specialized training, there can still be some variability due to farmer’s errors in sample collection and processing. To mitigate this, farmers can be provided with clear guidelines for sample collection and processing. Besides, the imaging device’s algorithm and calibration could potentially account for some variations arising from sample preparation.

Ensuring effective engagement with farmers and enhancing accessibility necessitates the integration of a robust database infrastructure. For seamless data management and retrieval, the proposed application requires a well-designed database. This database could be established either as a cloud-managed database (e.g., Snowflake warehouses, Amazon RDS, Google Cloud SQL, Microsoft Azure SQL Database, etc.) or a large stand-alone database such as PostgreSQL, MySQL Server, or Microsoft SQL Server. A cloud-managed database offers the advantage of scalability and remote accessibility, enabling stakeholders to access data and insights in real-time from anywhere. This architecture leverages cloud services to store, manage, and secure data, accommodating varying levels of demand without compromising performance. Conversely, a large stand-alone database offers localized data storage and management, catering to scenarios where connectivity might be limited. Such a solution ensures quick access to data within the immediate vicinity. For example, some agricultural businesses and farms deploy stand-alone servers to run farm management software, such as AgriWebb, Granular, and FarmLogs, etc. [46]. By incorporating either of these database approaches, the proposed technology can potentially bridge the gap between innovation and accessibility, supporting informed decision-making by farmers and stakeholders alike. Notably, the SMART NP application provides the feature of result sharing, which can be used to log the results in a device where the above two architectures are not feasible. Later, these logs can be retrieved to synchronize the data with analytical tools and programs.

In conclusion, the integration of smartphone imaging devices with advanced CTA methods offers a practical solution for resource-poor farmers. This technology empowers farmers to make informed decisions regarding nutrient management by accurately predicting soil NO_3_^−^ content and generating spatial variability maps. By adopting this approach, farmers can optimize their nutrient management strategies, increase productivity, reduce costs, and minimize environmental impacts. The proposed approach was not intended to provide a comprehensive assessment of all aspects of soil fertility but rather to offer a rapid and accessible means of evaluating one critical aspect—N availability—within the broader fertility context. The simplicity, accessibility, and cost-effectiveness of this technology make it a valuable tool for improving agricultural practices and supporting sustainable farming in resource-poor conditions. While the proposed approach may require certain sample preparation steps, more research is needed to develop a comprehensive portable soil testing solution that incorporates not only the imaging device but also other field sampling and processing attachments. This integrated approach will take into consideration the advancements in portable technology and existing solutions available in the market. The ultimate goal should be to create a user-friendly and efficient solution that minimizes the complexities associated with field testing procedures.

## 4. Conclusions

In this study, a smartphone-integrated imaging device was optimized and validated in collaboration with the SMART NP stand-alone mobile application, leveraging an advanced CTA-based color development method. This innovation represents a promising alternative to classic colorimetric approaches, offering rapid and precise estimation of soil NO_3_^−^ levels. The integration of this technology seamlessly empowers users with comparatively faster access to predicted outcomes, enabling prompt decision-making in resource management. Furthermore, the study showcased the capability of the device-predicted soil NO_3_^−^ data in conjunction with kriging interpolation for rapid evaluation of soil NO_3_^−^ spatial distribution at the regional level in Eastern India. The comparison with laboratory-derived data highlighted the device’s reliability and accuracy in predicting soil NO_3_^−^ levels. Overall, the smartphone-integrated imaging device, coupled with geostatistical interpolation, offers a comprehensive solution for rapid assessment of soil NO_3_^−^ spatial variability. With its practicality, affordability, and accuracy, this technology can become a valuable tool for farmers, agronomists, researchers, and environmental agencies, contributing to sustainable soil resource management and safeguarding human health. While some intricacies are still involved in sample collection and handling, this research underscores the feasibility of incorporating these steps within the scope of the technology’s practical implementation. With further advancements and implementation, this technology has the potential to revolutionize soil nutrient management practices and contribute to improved agricultural productivity and sustainability.

## Figures and Tables

**Table 1:** Comparison of the device performance with different smartphone cameras.

Smartphone	Camera (Megapixels)	Media	R^2^	Equation
HONOR-20i	24	Soil	0.98	NO_3_^−^ = 24.82 − 0.26x ^1^
Redmi 12 Pro	50		0.98	NO_3_^−^ = 231.75 − 2.34x
Vivo Y1S	13		0.90	NO_3_^−^ = 171.12 − 1.70x

^1^ x = average V-value.

**Table 2:** Comparison of the performance for measuring analytes in soil and water using smartphone-based methods, as reported by researchers.

Authors	Media	Analyte	R^2^	Equation
Present study	Soil	NO_3_^−^	0.98	NO_3_^−^ = 24.82 − 0.26x ^1^
[16]		NO_3_^−^	0.98	NO_3_^−^ = 16.94 − 0.17x
[16]		PO_4_^3−^	0.96	PO_4_^3−^ = 2.49 − 0.026x
[14]		PO_4_^3−^	0.99	PO_4_^3−^ = 0.3930 × exp(−x/1.854) + 0.3978
[16]	Water	NO_3_^−^	0.97	NO_3_^−^ = 11.87 − 0.12x
[16]		PO_4_^3−^	0.98	PO_4_^3−^ = 45.74 − 0.49x
[14]		PO_4_^3−^	0.99	PO_4_^3−^ = 0.3930 × exp(−x/1.854) + 0.3978
[13]		Cl	0.99	Cl = 86.008z ^5,2^ − 359.04z ^4^ + 556.14z ^3^ − 402.96z ^2^ + 135.15z − 15.804

^1^ x = V-value. ^2^ z= color ratio. The superscript numbers (3–5) indicate power functions.

**Table 3:** Regression functions used for kriged datasets variogram models using device predicted and laboratory-measured soil NO_3_^−^.

Kriging Parameters	Device-Predicted Values	Laboratory-Measured Values
Variogram model	Gaussian	Gaussian
Nugget	204.84	222.64
Partial Sill	47.65	39.68
Nugget/Sill Ratio	0.81	0.85
Regression equation	Soil NO_3_^−^ = 0.51x ^1^ + 15.12	Soil NO_3_^−^ = 0.49x + 16.35
RMSE	1.07	1.07

^1^ x = device-predicted/laboratory soil NO_3_^−^ concentration.

## Data Availability

The data presented in this study are available on request from the corresponding author.
